# A Model Hospital

**Published:** 1901-12-21

**Authors:** 


### A MODEL HOSPITAL.

### BOLINGBROKE HOSPITAL: OPENING THE NEW OUT-PATIENTS' DEPARTMENT.

To leap suddenly into fame is perhaps as unusual among hospitals as among individuals; yet such a fate befell the small Bolingbroke Hospital, Wandsworth, on Wednesday, December 11th, on the occasion of the opening of the new wing for out-patients. The ceremony was performed by Mr. Thomas Bryant, Surgeon-in-Ordinary to the King, and Consulting Surgeon to Guy's Hospital.

After preliminary prayer by the Rev. A. Gordon Ward, vicar of the parish, and a historical sketch of the hospital by Canon Erskine Clarke, Mr. Bryant delivered an interesting address, which will be found in full in the *Lancet* of December 14th, 1901.

A vote of thanks to Mr. Bryant was proposed by Mr. J. Mackrell, and seconded by Mr. Hennesley, of the Metropolitan Asylums Board, to which Mr. Bryant replied that the twelve years during which he had been working at the Bolingbroke Hospital had been very valuable to him; he was very glad if they had been useful to others too. This object-lesson ought to be repeated in all large suburbs of the metropolis, and as a type of a provincial hospital it should be copied, while if some of its principles were followed by the larger hospitals some objections to them as institutions might be done away with.

A vote of thanks to Canon Erskine Clarke was proposed by the Chairman of the Wandsworth Board of Guardians. This was seconded by Sir Henry Burdett, who, speaking as one deeply interested in hospitals, said he was afraid if he kept silence, what he felt to be the right object of the meeting might be lost sight of. He thought that the meeting should have only one end, and that was, that Wandsworth should have a complete hospital, on the lines of the plan hanging on the wall.[1] If he understood anything about the feeling of those who supported hospitals in London, he believed that this hospital would be the first of the present century to be built, for it was going in the right direction. This was an occasion that should be made the most of for good. He had nothing to do with Wandsworth, but he had been interested in this hospital before it was actually started, and he knew all about it. He thought if we looked at ourselves as a nation we could not fail to realise that there was a weakening of the fibre. *Punch* had illustrated this in a picture of a young man, after vaccination, swathed in bandages, ringing a handbell, and saying to the passers by, "Keep to the left: don't touch." London trade was suffering because of small-pox, and the authorities seemed to be using that fact to induce people to be vaccinated. People were praising the nurses for attending cases of small-pox, whereas doctors and nurses knew that they might live quite close to the infection and that no harm need come of it. This weakening of the fibre of the people was a very great danger, and he was not charging the young with it, but those who had attained seniority. The "Tommies," for example, had shown wonderful fibre in endurance and courage. This had much to do with the question before them. The hospitals as they were at present conducted tended to weaken the fibre of the people; and for that reason, the knowledge that they had a hospital in Wandsworth, working quietly and wisely under the right system, was a blessing for which every thinking Londoner might well thank God and take courage. More hospitals on this system were wanted. The bad habit of following old plans ought to be broken. Why were not all our hospitals conducted on this plan? There was another fact. In Wandsworth the majority of the people belonged to the working classes; and the mere circumstance that they had succeeded, partly through the co-operation of the medical profession, and still more through that of the working classes themselves, proved that the faults and abuses of the present system were not the fault of the working classes, but of those who conducted the hospitals. He proposed that Dr. Bryant's address be widely circulated; and just as something like 70 cottage hospitals were built as the result of an address, so much good ought to follow Dr. Bryant's address. If it could be made perfectly clear that they wanted a hospital for the hundreds and thousands of poor who had shown that they could give something towards its support, large numbers of people would send contributions, and this hospital would be a pioneer, to reform, once for all, those abuses of charity which now attended the ministrations to the sick. The facts had been buried, but he had dug them up. He believed that the cost of each wing of this hospital was estimated at £20,000; and he could undertake to say that they could be built for that sum, and on a more generous scale than was shown in the plans. Many rich men would spend that sum in gifts; and he would like it to go forth that there was no collection of gifts on which that amount of money could be more wisely spent than on this hospital. People would not go on subscribing these large sums unless they were sure that the old bad principles were going to be rooted out; and if these facts could be brought home to those who supported hospitals, before the year was out, there would be no difficulty in placing in the hands of the governors of the Bolingbroke Hospital the whole of the £60,000 to give the working people of Battersea the right to build up the missing link which had been sought for for years. It would make medical relief cease to be the aid to pauperism that it now was. If there was one test of good work, it was its being done quietly and almost unnoticed, and if ever there was a work founded upon the right principle, of the greatest good for the greatest number, it was that being done by the Wandsworth Hospital, and it was owing to Canon Erskine Clarke, who founded it.

Canon Clarke, in replying, said he did not much care for votes of thanks, but he was quite happy this time, since he had evoked from Sir Henry Burdett an expression of those deep principles which, quite unconscious of their wider purport, he had been trying to work out.

The meeting, which was held in the new white-tiled out-patients department, then terminated.

[1] The plan was reproduced in The Hospital for Nov. 30th.

